# The cost of illness and burden of suicide and suicide attempts in France

**DOI:** 10.1186/s12888-024-05632-3

**Published:** 2024-03-19

**Authors:** Laeticia Blampain Segar, Charles Laidi, Ophélia Godin, Philippe Courtet, Guillaume Vaiva, Marion Leboyer, Isabelle Durand-Zaleski

**Affiliations:** 1 Fondation FondaMental, Créteil, F-94010 France; 2 AP-HP Health Economics Research Unit, Hôtel-Dieu Hospital, INSERM UMR 1153 CRESS, Paris, France; 3 IMRB, Translational Neuro-Psychiatry, Univ Paris Est Créteil (UPEC), INSERM U955, Créteil, F-94010 France; 4 AP-HP, Département Médico-Universitaire de Psychiatrie et d’Addictologie (DMU IMPACT), Fédération Hospitalo-Universitaire de Médecine de Précision en Psychiatrie (FHU ADAPT), Hôpitaux Universitaire Henri Mondor, Créteil, F94010 France; 5 Child Mind Institute, New York, USA; 6 Univ. Lille, Inserm, CHU Lille, U1172-LilNCog-Lille Neuroscience & Cognition, Centre de Ressources et Résilience pour les Psychotraumatismes (Cn2r Lille Paris), Lille, F-59000 France; 7 Université Paris Est Créteil, Creteil, France

**Keywords:** Suicide, Suicide attempts, Health economics, Cost, Death, Disability

## Abstract

**Background:**

With 11,558 deaths and 200,000 suicide attempts in 2019, France is among the European countries most affected. The aim of this study was to determine the costs and burden of suicides and suicide attempts in France (population 67 million).

**Methods:**

We estimated direct costs, comprising healthcare, as well as post-mortem costs including autopsy, body removal, funeral expenses, police intervention and support groups; indirect costs comprised lost productivity, daily allowances; the burden of disease calculations used a monetary value for death and disability based on incidence data. Data was obtained from the national statistics, health and social care database, registries, global burden of disease, supplemented by expert opinion. We combined top down and bottom up approaches.

**Results:**

The total costs and burden of suicides and suicide attempts was estimated at €18.5 billion and €5.4 billion, respectively. Direct costs were €566 million and €75 million; indirect costs were €3.8 billion and €3.5 billion; monetary value for death and disability was €14.6 billion and €1.3. The monetary value for death and disability represented 79.1% and 24.8% of total costs for suicide and suicide attempt respectively.

Some costs were based upon expert opinion, caregivers’ burden was not counted and pre COVID data only is reported.

**Conclusions:**

In France, the total cost and burden of suicides and suicide attempts was several billion €, suggesting major potential savings from public health interventions.

**Supplementary Information:**

The online version contains supplementary material available at 10.1186/s12888-024-05632-3.

## Background

Suicide is one of the primary causes of mortality worldwide [1]. The projected 50% increase in incidence by the year 2030, would make it the 12th leading cause of death [1, 2]. Its estimated annual mortality rate is 700,000 individuals, or one out of every 100 deaths in the world. It is also the fourth leading cause of death among individuals aged 15 to 29 years, trailing only motor vehicle accidents, tuberculosis, and interpersonal violence [1]. On a global scale, the incidence of suicides is higher among men, with rates of 5.4 per 100,000 and 12.6 per 100,000, in women and men respectively. In high-income countries, the suicide rates among men are typically higher, with a rate of 16.5 per 100,000. By contrast, among women, suicide rates are highest in lower-middle-income countries, with a rate of 7.1 per 100,000 [3, 4]. In the USA, the average cost of one suicide was estimated to be $1.06 million [5]. In 2019, the French suicide mortality rate remained significantly higher than the European Union average, at 13.07 per 100,000 inhabitants vs. 10.15 per 100,000 [6]. 

The COVID-19 pandemic had a considerable effect on the psychological state of millions of people around the world [7]. Many experts have expressed concern regarding the mental, economic, behavioral, and psychosocial problems associated with the COVID-19 pandemic that may contribute to increased suicidal ideation, suicide attempt, and self-harm [8]. The literature on the cost of suicide remains scarce, the more recent publications considered suicide as an outcome of depression and treatment resistant depression [9, 10]. There is a need to document the disease burden and cost of illness to support the public policies focusing on suicide prevention as a health goal. In the UK, the analysis of costs/savings predicted that the additional costs of training general practioners on the identification and prevention of suicidal risk were offset at one year and the intervention was cost saving by a very large margin when productivity savings were included [11, 12]. In France, the establishment of the national emergency hotline for suicide prevention (3114) has been a key gateway for suicide prevention programs across the country. In 2021, the hotline received 85,000 calls, averaging nearly 450 calls per day [13]. This initiative cost €21 million [14]. The VigilanS system, deployed in 17 regions and 92 departments, is a call and alert system that coordinates a network of healthcare professionals around individuals who have attempted suicide to maintain contact with them. The annual cost of this system was estimated at approximately €486,000 per region, or nearly €8.4 million per year. The deployment of VigilanS from 2015 to 2017 was associated with a 38–41% relative reduction in the risk of recurrent suicidal behavior (emergency room visits, hospitalizations for suicide attempts, or suicide deaths) within the 12 months following a suicide attempt among patients enrolled in VigilanS, compared to the group of patients not exposed to the program. The sustainability of such programs requires evidence that they provide not only better health outcomes but also some return on investment.

The objective of this study was to estimate the cost and burden of suicide attempt and suicides in 2019 prior to COVID in France. We estimated the direct medical and non-medical costs, the indirect costs, and attributed a monetary value to death and disability. For easier international comparisons, we averaged the costs over the total population of France.

### Populations and methods

This is an incidence-based study, estimating the cost and burden of suicide and suicide attempts from a societal perspective.

The populations considered for the cost calculations were the completed suicides and the suicide attempts over a one-year period, their respective numbers where obtained from the Ministry of health for the year 2019.

### Epidemiological approach

The Global Health Data Exchange ‘GHDx) and the directorate of statistics at the French Ministry of Health (DREES) reported the total number of suicides and suicide attempts with an intervention for 2019: 11,558 suicides and 200,000 suicide attempts [15, 16]. This latter number is a rounded up estimate because some suicide attempts did not result in any encounter with the health care system.

### Costing approach

We combined top down and bottom up approaches depending on the data available; we used for the most part public information and retrospective data, supplemented when necessary by expert opinion. We used an incidence approach and a one-year time horizon to estimate recurring health care and non-healthcare direct costs. To estimate the productivity losses attributable to suicide, we used the cumulated number of life years lost between the average age of suicide and 65 years.

### Identification of resource use

Direct healthcare costs included all resources used in the prevention, care and follow up of suicide attempts and suicides. Direct non healthcare costs included forensic and police interventions, post mortem services, funeral services, support groups. We included disability allowances and loss of productivity in the calculation of indirect costs. We attributed a monetary value to the loss of health and life years.

The calculations did not consider a counterfactual scenario with suicide prevention and with no deaths by suicide and included costs that may be avoidable by preventive measures as well as unavoidable costs.

#### Health and social care costs

The French national healthcare database records hospital stays in acute, psychiatric and rehabilitation units; we extracted index admissions and subsequent admissions during 2019 using diagnostic codes of self harm [17]. Self-harm is defined by the International Statistical Classification of Diseases and Related Health Problems 10th revision (ICD-10) as an intentional poisoning or traumatic injury inflicted by an individual upon themselves. These events are classified within codes X60 to X84 in the sub-chapter “self-inflicted injuries”, which encompasses external causes of mortality and morbidity. The codes are presented in Supplementary material 1. For surviving patients, one-year admissions were compiled using record linkage. All suicides were identified by the discharge mode “death”.

The emergency department (ED) surveillance database (OSCOUR) controlled by “Santé Publique France”, collects medical diagnoses on hospital ED visits. In 2019, the database covered 94% of ED visits in the country [18]. It provided the total number of ED visits for suicide and suicide attempts and mode of transportation.

The general practitioners’ surveillance Sentinel network (Réseau Sentinelle), collects epidemiological data from volunteer physicians to analyse the frequency and distribution of different diseases including suicide attempts. We used it to estimate the number of annual consultations using the % of consultations before a suicide attempts and the % of referrals to a psychiatrist combined with the total of suicides and suicide attempts [19]. National guidelines post suicide attempts recommend systematic referral to a psychiatrist and we assumed full adherence.

The annual expenditures and revenues report published by the French National Health Insurance Fund (CNAM) estimated the 2019 cost associated with psychotropic drug consumption for psychiatric illnesses [20]. This cost was used to calculate the average drug cost following a suicide attempt, with the assumption that the cost would be the same for suicide attempts as for all beneficiaries.

#### Non healthcare costs

Each suicide requires police intervention. In France, not all suicides are subject to a forensic investigation, despite the European recommendation to perform an autopsy in cases of violent death, especially in cases of suspected suicide. In an epidemiological study of suicide victims examined by a forensic institute in Angers from 2014 to 2016, 43% of suicides were autopsied [21]. We assumed that each autopsied suicide case underwent an imaging and toxicology examination in addition to external and internal examination.

We used the governmental Finance Act for 2019 to identify the total funding for support groups involved in suicide prevention, and the report of SOS Amitié (friendship) to estimate the respective proportions of calls for suicides and suicide attempts [22]. 

#### Indirect costs

Indirect costs calculations used the number of full-time equivalents generated by sick leave obtained from health insurance data, [23] and literature estimates for the proportion of individuals on sick leave for depression [24, 25] We assumed that the duration of sick leave for suicide attempts was the same as the average duration for patients with psychiatric disorders.

The Global Health Data Exchange (GHDx) database provided the number of years lost due to disability and premature death from suicide and suicide attempts. Both are associated with disability-adjusted life years (DALYs) a time-based measure that combines years of life lost due to premature mortality (YLLs) and years of healthy life lost due to disability (YLDs). The number of years lost per patient was estimated from the total Years of Life Lost (YLL) and the number of suicides obtained from the Global Burden of Disease (GBD). The productivity losses attributable to premature death were estimated by subtracting the average age of suicide from the official retirement age of 65 years.

The amount of DALY attributed to suicide attempts and suicide was extracted from the GDB using 2019 data for self-harm in France. The burden for suicide was estimated by the number of life years lost and the burden for suicide attempts was estimated from the remaining DALYs (by subtracting the YLLs from the total DALYs) [15]. 

### Measurement and valuation

The measurement and valuation method was adapted to the type of data available. Whenever possible we used primary data e.g. discharge data with diagnoses for hospital costs, actual number of consultations and ER visits. When direct identification of resource use for suicide and suicide attempts was impossible, we used average resource use for psychiatric illnesses as a proxy and multiplied unit costs by the number or suicides or suicide attempts whenever appropriate.

#### Direct healthcare costs

##### Hospital

The cost of the hospital stay in acute care and psychiatry was valued using the national cost study, adjusted for the actual length of stay and intensive care unit for each patient and the stratified daily cost by Diagnosis-Related Group (DRG) and a per diem for psychiatry.

##### Emergency costs

The average €227 cost of ED visits was obtained from the report to the Senate [26].

##### Transport costs

The average cost of transport is presented in the Supplementary material 2.

##### Outpatient costs

We computed the cost of consultations to GPs and referrals to psychiatrists, both pre- and post-suicide attempts as they pertained to the same event using the average cost of a psychiatrist consultation.

Additionally, we calculated the cost of psychotropic drugs consumption by using the total expenditure on drugs attributed to psychiatric illnesses and the number of patients calculated by the health insurance system [20].

#### Direct non healthcare costs

##### Post-mortem costs (autopsies and forensic intervention)

The average total cost of an autopsy was estimated to be €2,604 [27–29]. Verification of the extinction of life by a physician is systematically carried out, and the cost of body removal was estimated at €57.50, based on the rate applied since January 1, 2011 [30]. Similarly, the cost of police intervention for each suicide was calculated. Funeral expenses were also included for each death, and the average funeral cost was calculated from a weighted average of cremation and burial. In 2019, 39% of the deceased had a cremation, and 61% were buried [31]. The average costs of cremation and burial were estimated from surveys, the average cost of a funeral was estimated to be €4,322 [32, 33]. Unit costs are available in the Supplementary material 3.

### Support groups costs

These organizations are government-funded, and their global budget was obtained from the database of funds allocated to nonprofit organizations [34]. 

#### Indirect costs

Loss of productivity was calculated using the human capital approach. The cost premature death was measured by the loss of productivity, discounted to its present value. We assumed that individuals work until an average age of 65, using the available average age of decedents from the GHDx database [15]. The years of work lost were valued using the €36,185 (2019) per capita GDP [35]. We also estimated the daily allowances provided as income compensation post suicide attempts by the social health insurance, which we extrapolated to the total number of suicide attempts. Reporting both income compensations and loss of productivity made sense because they affect different stakeholders, however adding those up resulted in double counting. We paid special attention to this point in the presentation of the results.

### Monetary value of death and disability

The cost of DALYs was calculated based on the Global Burden of Disease and Injury (GBD) report. We valued one year of life lost based on the 2019 GPD per capita.

A summary table of data sources, methods of identification and calculation and costing approach is presented in the online supplements Supplementary material 4.

### Sensitivity analysis

The Ministry of Health routinely adjusts for the 10% underestimate in the national center for Epidemiology on mortality causes (Centre d’Epidemiologie sur les causes medicales de décès or CepiDC) data. In order to assess this effect, we reanalyzed the total cost using the number of suicides identified by CepiDC for 2019 adjusted by the underestimation [36, 37]. 

Different discount rates (0% and 4%) were applied to assess the costs associated with premature deaths and productivity losses resulting from suicides.

The valuation of the costs associated with YLLs and YLDs was recalculated using the value of a life year (VOLY) estimated at 45,000 euros [8]. 

### Statistical analysis

Average costs for each cost category were calculated per patient, considering 11,558 completed suicides and 200,000 attempted suicides. These values were then projected on a per capita basis (67 million inhabitants) to facilitate international data comparison. When the cost of a DRG was not available in the National cost study, we calculated the average cost of the closest DRGs available based on the legal status of the establishments, and applied it to the missing costs. All data were analyzed using R software (Version 4.1).

## Results

### Direct healthcare costs

In France, a total of 84,014 admissions in acute care following a suicide attempt were observed for 72,076 patients, with 990 deaths resulting from these attempts. Among admissions for suicide attempt, 62% were women and among admissions for suicide, 67% were men. 17,413 patients were hospitalized in psychiatric hospitals for suicide attempt and suicides, with 26,053 stays (including repeat admissions) during the year. Most admissions were inpatient stay, with the cost of full inpatient care totaling €184 million and emergency care €13 million. Rehabilitation amounted to 974 stays (including repeat admissions) for 931 patients, with a cost of €13.2 million.

#### Emergency department and transport costs

ED visits for suicide attempts and suicide totaled 97,263; suicide attempts accounted for the majority of the overall cost (99%) of €22.08 million. The transportation cost associated with ED visits was €58 million.

#### Outpatient costs

The cost of consultations prior to suicide attempt was €10.31 million, while the cost of consultations following a suicide attempt was estimated at €9.35 million. Consultations preceding completed suicides were estimated at €0.50 million. Finally, the cost of psychotropic drugs consumption after suicide attempt was evaluated at €75.07 million.

The total healthcare cost for suicide attempts and suicides was estimated at €190 million.

### Post-mortem costs

We estimated the average cost of an autopsy at €2,604. Autopsies were conducted for 43% of suicide deaths, resulting in a cost of €13 million. Body removal was performed post-mortem and valued at €57.50 per removal. The average cost of police intervention was estimated at €278 per suicide. Funeral costs for all suicide deaths were €4,322.4 or 75% of the total post-mortem cost.

### Support groups costs

The budget allocated for supporting groups that focus on suicide and self-harm was estimated to be €0.28 million. Among the individuals seeking assistance from these associations, approximately 66% displayed suicidal behavior or had previously attempted suicide, while the remaining 34% sought support for grief-related issues.

#### Loss of productivity and daily allowance

The individual productivity loss due to suicide was estimated at 9.07 years. Additionally, 322,022 sick leaves were recorded due to depressive disorders, with an average duration of 81.4 days. The number of full-time-equivalent per year was estimated at 71,811 representing a total loss of €7,243 million. Daily allowances represented €69 million, or 2% of the indirect costs of suicide attempts, a money transfer rather than a cost.

#### Death and disability

The GHDx data allowed us to estimate that suicides accounted for 404,172 YLLs while suicide attempts resulted in 37,200 YLDs for suicide attempt and a total of 16 Bn€.

#### Total cost of illness and burden of suicide and suicide attempts in France

The economic impact of suicides and suicide attempts totaled €24 billion, the cost breakdown is shown in Table 1 (total costs) and Table 2 (average costs). Considering the total French population of 67 million, the direct cost was estimated to be €1.12 per capita for suicide and €8.40 per capita for suicide attempt. The indirect cost was estimated at €56 and €52 per capita for suicide and suicide attempt respectively, and the intangible cost was €217 and €20 per capita for suicide and suicide attempt respectively. The total per capita cost for suicide was estimated to be €274 and €81for suicide attempt, a weighted average of €355 per capita of which 9.6€ were direct costs.

**Table 1:** Direct, indirect costs and death and disability costs related to suicide and suicide attempt in France in 2019 (in millions euros)

Cost of suicide and suicide attempts	Suicide *N* = 11,558	Suicide attempts *N* = 200,000	Total
**Direct cost**	**75**	**566**	**641**
**Healthcare**	**8**	**566**	**574**
Acute care	7	183	190
Rehabilitation	0.13	11	11
Psychiatry	0.19	197	197
Emergency department	0.01	22	22
Transport	0.01	58	58
Outpatient care	0.50	95	96
**Post-mortem cost**	**67**	**0**	**67**
Autopsy	13	0	13
Body removal	0.66	0	0.66
Police	3.5	0	3.5
Funeral expenses	50	0	50
**Support groups**	**0.10**	**0.18**	**0.28**
**Indirect cost**	**3,793**	**3,519**	**7,313**
Daily allowances	0	69	69
Productivity loss	3,793	3,450	7,243
**Death and disability monetary value**	**14,625**	**1,346**	**15,971**
**Total**	**18,493**	**5,431**	**23,925**

Note: We summed the direct, indirect costs and the monetary value of death and disability to provide a summary figure. This figure however includes a double counting for daily allowances and productivity losses; combining costs, losses and monetary value of death and disability is not recommended, although commonly undertaken for advocacy purposes

**Table 2:** The average cost of suicide attempt and suicide in €, France 2019

	Suicide *N* = 11,558	Suicide attempts *N* = 200,000
**Direct cost**	**6,506**	**2,831**
**Healthcare**	698	2,830
Post mortem	5,799	0
Support groups	9	1
**Indirect cost**	**328,198**	**17,595**
Allowances	0	342
Productivity loss	328,198	17,252
**Death and disability monetary value**	**1,265,353**	**6,730**
**Total**	1,600,057	27,156

### Sensitivity analysis

Data from CépiDc identified 8,632 suicide deaths in 2019. However, it is estimated that national data is underestimated by 10% due to deaths where the cause is undetermined or forensic examination results were not reported. After adjusting this underestimation, the number of suicide deaths in 2019 was 9,495. The average direct cost of suicides was €6,651 per patient, while the indirect cost was €328,233 per deceased patient. Additionally, the cost of death and disability was €1,540,279 per patient.

Using the VOLY valuation increased the monetary burden of suicides and suicide attempts to €18.19 billion and €1.67 billion euros.

Using a 4% discount rate reduced the productivity losses to €3.2 bn, a difference of €0.6 bn, and the value of death and disability to €8.1 bn.

## Discussion

To our knowledge, this study is the first to assess the cost of suicides and suicide attempts and the monetary value of death and disability in France prior to the COVID-19 pandemic. Our comprehensive assessment of the costs associated with suicide and suicide attempts found a summary figure of 24 billion euros in 2019, including direct costs (0.4% for suicides and 10.4% for suicide attempts), indirect costs (20.5% for suicide and 64.8% for suicide attempts), disability, and death (79.1% for suicide and 24.8% for suicide attempts). The average cost per capita was €355.

Our results contribute to existing knowledge for two main reasons. First, this topic appears to be under-researched, with no systematic review published to date, and the only international study on the cost of suicide focused on youth deaths in highly developed countries [38]. International comparisons are challenging because of differences in methodological approaches and data sources that encompass various cost components. Second, suicide and suicide attempts suffer from limited availability of data. In France, the estimation of suicidal behaviors is primarily based on the number of individuals hospitalized for these reasons. However, practitioners often code suicide attempt (“self-harm” in the ICD-10 classification) poorly upon hospital discharge, coding instead the primary psychiatric disorder [39]. Furthermore, a large proportion of individuals who attempt suicide and may only visit the emergency department for follow-up care. Consequently, the reasons behind their ED visits are often not properly recorded or centralized [40]. This suggests that suicide attempts may go unreported, resulting in an underestimation of the overall economic costs of suicide and suicidal behavior.

Despite methodological differences, our findings were consistent with those of other countries when converted to per capita costs in euros for 2019 and adjusted for population differences.

In the USA, direct costs of suicides were estimated at €0.70 per capita and €34.89 per capita for suicide attempt. Indirect costs were estimated at €8.60 per capita for suicides. Finally, intangible costs for suicide and suicide attempt were €1,282 and €30.46 per capita, respectively, for the year 2019. This represents a total cost of €1283 and €73.95 per capita for suicides and suicide attempts [41]. In New-Zealand, the direct and indirect cost was €77 per suicide and €13 per suicide attempt for the year 2019 [42]. In Ireland, the cost of suicide, considering direct, indirect, and intangible costs, was €302 per capita [43]. In Netherlands, the cost of a completed suicide were estimated €1,769,801, equivalent to €0.11 per capita [44]. 

An investigation into the costs of suicide and suicide attempt following the COVID-19 pandemic would be relevant. Mixed results regarding the impact of the pandemic on hospital consultations for self-harm have been reported, with some studies reported a decrease in self-harm cases, while others reported no change or an increase during certain phases of the pandemic confinement period [45–47]. 

Cost of illness and burden of disease studies can be used to estimate the return on investment from preventive measures. The main strength of this study is its scope. Indirect and intangible costs are frequently disregarded in economic and scientific research due to the intricate nature of their quantification and the challenge in ascribing a precise monetary value to them. Omitting these costs may result in an underestimation of overall expenses and misallocation of resources [48]. 

### Limitations

The total summary figure of €24 billion includes double counting, merges the social and public sector perspectives, and adds the monetary value of death and disability which is not recommended in Drummond et al. [49] The double counting concerns daily allowances and sick leaves which are transfers and not costs, and productivity losses, reported as costs. This accounting practice stems from the diverse perspectives of the stakeholders involved: while the allowances are actual payments from the budgets of social insurance and complementary health insurance, the productivity losses reflect the expected reduction in economic output. Nevertheless, as the allowances are intended to compensate for patients’ income loss, they effectively result in a transfer of resources from one sector to another. We chose to report a global summary figure in order to allow comparisons with other studies in mental health or other disease areas such as the recent cost of addictions in France which used the same approach [50]. The disaggregated presentation by type of costs allows the identification of costs by stakeholder.

The suicide attempts were identified from national statistics which rely on resource use from the healthcare system. The less severe cases that did not require any medical intervention were ignored. We were unable to account for the productivity loss resulting from presentism, when employees are physically present but inactive or unproductive, which is not unlikely in a population of depressed individuals. The rate of unemployment in this population was not taken into account, which leads to an overestimation of indirect costs. We did not estimate the extra medical needs, absence from work and loss of quality of life for caregivers due to the lack of data.

Finally, we recommend that future research on the economic cost of suicide and self-harm systematically take into account all components, including direct, indirect, and intangible costs, as well as disabilities and deaths that occurred in the most recent year, as we attempted to do in this study to establish more reliable comparisons.

## Conclusion

This study provides the first assessment of the economic burden of suicides and suicide attempts in France for the year 2019. Further research is necessary to examine the potential impact of the COVID-19 pandemic on these economic costs. These results should alert policymakers about the importance of improving suicide prevention and encourage research on this subject to address this avoidable burden.

### Supplementary Information

**Supplementary Material 1.**

## Data Availability

The data that support the findings of this study are available from the corresponding author, upon reasonable request. The costs to the healthcare system are available at Les propositions de l’Assurance Maladie pour 2024 | L’Assurance Maladie (ameli.fr).
