# Supplementary material for: The cost of illness and burden of suicide and suicide attempts in France
Source: BMC Psychiatry. 2024 Mar 19;24:215. doi: 10.1186/s12888-024-05632-3 (PMC10953174; doi:10.1186/s12888-024-05632-3)
Supplement: Supplementary file 1 — Supplementary Material 1. [file 12888_2024_5632_MOESM1_ESM.docx]

**Supplementary material:**

**Supplementary material 1: ICD-10 codes for suicides and suicide attempts**

The codes used correspond to deliberate self-inflicted intoxication or traumatic injury. The stays were selected when the primary diagnosis was the consequence of the suicidal act (traumatic injuries, poisoning, etc.) and was coded as 'Z915', or when an associated diagnosis in the range X60 to X84, Z915, or Y870 was identified. Duplicate data was excluded. Similarly, some stays were excluded when: Only a diagnosis of alcohol self-intoxication (x65) or drug self-intoxication was mentioned (X62). Only a diagnosis of a history of SA was mentioned (Z915) without a diagnosis of drug poisoning (T36-T50); A combination of the first three diagnosis was mentioned without drug poisoning ; only a diagnosis of sequelae of SA was mentioned (Y870) and only a diagnosis of drug self-intoxication was mentioned (X60-X61, X63-X64) without a diagnosis of drug poisoning. Codes have been combined and are presented in Table 1.

Table 1 : identification of ICD 10 code for SA

| Code ICD-10 | Diagnosis |
| --- | --- |
| X60 to X64 | Self-poisoning by drugs; |
| X65 to X69 | Self-poisoning by other substances (alcohol, solvents, gases, pesticides, chemicals); |
| X70 | Self-inflicted injury by hanging, strangulation, suffocation; |
| X71 | Self-inflicted injury by drowning, submersion; |
| X72 to X74 | Self-inflicted injury by firearms; |
| X75 to X77 | Self-inflicted injury by exposure to smoke, flames, and gases; |
| X78 to X79 | Self-inflicted injury by sharp object; |
| X80 | Self-inflicted injury by jumping from a high place; |
| X81 to X82 | Self-inflicted injury by intentional collision; |
| X83 to X84 | Self-inflicted injury by unspecified means. |
| T36 to T50 | Drugs poisoning diagnosis |
| Y870 | Sequelae of Self-Inflicted Injury |
| Z915 | Personal history of self-inflicted injuries |

**Supplementary material 2: The average cost of transportation**

| Methods of transportation | Average cost per intervention (€) | Sources |
| --- | --- | --- |
| Public or private ambulance | 94,78 | ^65^ |
| Police | 299,74 | Expert opinion |
| Helicopter | 2 641,41 | ^66,67^ |
| Personal transport | 25,00 | ^65^ |
| Mobile emergency and intensive care service (SMUR) | 2 184,25 | ^66,67^ |
| victim assistance and rescue vehicle (VSAV) | 1 079,30 | ^68^ |

**Supplementary material 3: The average cost of post mortem**

| Service | Unit of measurement | Estimated amount (€) |
| --- | --- | --- |
| Autopsy | Per suicide | 1119,72 |
| Body removal | Per suicide | 57,50 |
| Police | Per suicide | 299,74 |
| Funeral expense | Per suicide | 4322,40 |
| Total |  | 5799,36 |

**Supplementary material 4: summary table of the identification and calculation of costs**

| **item** | **Identification** | **Calculation** | **Source** |
| --- | --- | --- | --- |
| Hospital costs : acute care, psychiatric care and rehabilitation care | Primary and secondary diagnoses  National hospital discharge database with one-year record linkage at the patient level | Full DRG cost for acute care and rehabilitation care for all patients identified in the database  Per diem x length of stay for psychiatric care for all patients identified in the database | ATIH |
| Emergency care | Suicide attempts as an entry diagnosis | Number of suicide attempts in the OSCOUR database x unit cost of ER | OSCOUR network |
| Transportation | Suicide attempts as an entry diagnosis and mode of transportation (ambulance, police, helicopter) | Number of transportation x unit cost for each type of transportation | OSCOUR network |
| Consultations | % of patients consulting before a suicide attempt, % of patients referred to a psychiatrist  Post suicide attempts we assumed that all patients (200,000) were referred to a psychiatrist | Number of suicides and suicide attempts x % of patients with GP visits and % of patients referred to a psychiatrist x total number of suicide and suicide attempts form the national statistics in 2019 x unit costs of consultations with GPs and psychiatrists | Sentinelle network of general practitioners  And national guidelines for care pathway post suicide attempt |
| Psychotropic drugs | Average drug cost per patient with an identified psychiatric diagnosis (any diagnosis) | Average drug cost x 200,000 | Social health insurance yearly report for 2019 |
| Autopsy | % of deaths being autopsied in France (all deaths) | Unit costs of an autopsy x number of suicides x % of deaths autopsied | Epidemiological study by the forensic department in Angers |
| Forensic intervention and police intervention (systematic for any violent death) | All suicides (legal requirement) | Unit cost of a forensic and police intervention x total number of suicides | National statistics on the number of suicides in 2019  Expert opinion on the duration of the intervention, the number and type of officers involved |
| Support groups | 9 support groups involved in suicide prevention received public funding | Full amount of the state funding x % for suicide and suicidal ideation | Finance Act for 2019  SOS Amitié to identify the respective proportions of calls for suicide and suicide attempts |
| Funeral expenses | Weighted average cost of burial and cremation | Average cost x number of suicides | Surveys from a French consumer group (Que Choisir) |
| Lost productivity | Suicides: total number and average age  Suicide attempts: we assumed that the average number of days off work would be the same as for other psychiatric diagnoses and estimated an average from the social health insurance data | Suicides: number of suicides x years lost (65 – average age) x per capita GDP  Suicide attempts: number of days off work for depression x per capita GDP | GHDx and national statistics  Social health insurance yearly report for 2019 |
| Income compensation (daily allowances) | Suicide attempts: same hypotheses as above | Number of days off work x average daily income compensation x200,000 | Social health insurance yearly report for 2019 |
| Burden of disease | Total DALYs from self-harm in France for 2019 | Total DALYs x per capita GDP | GHDx |

ATIH: agency for information on hospital care

DALY: disability adjusted life year

DRG: diagnosis related group

GHDx: Global Health Data Exchange

GDP: gross domestic product

OSCOUR network : coordinated observation of emergency care
